# How Behavioral Branding Affects Brand Equity

**DOI:** 10.3389/fpsyg.2022.904736

**Published:** 2022-07-08

**Authors:** Theo Lieven

**Affiliations:** Institute for Marketing and Customer Insight, University of St. Gallen, St. Gallen, Switzerland

**Keywords:** brand personality, salesperson’s personality, brand equity, behavioral branding, sales encounter

## Abstract

Maintaining and increasing brand equity is the top priority for most brand managers. This includes not only the areas of public relations and advertising, but also the way in which sales staff communicates regarding the brand. According to behavioral branding, the brand should be strengthened by the brand fit of the employees. To date, research and practice have developed more intuitive and heuristic methods for evaluating employee behavior and its impact on the brand. In this article, behavior will be operationalized and measured by personality and sales encounter experience. The method is based on Heider’s balance theory explaining the occurrence of cognitive dissonance in case of unbalanced states in triads, here the brand, the customer, and the salesperson. Findings show how discrepancies in personal behavior led to discrepancies in brand equities before and after the sales encounter.

## Introduction

Sales employees are involved in sales encounters with their clients. These encounters and the resulting relationships have been the subject of numerous studies (Bitner, 1990; Babin et al., 1999; Piercy et al., 2001; Darian et al., 2005; Bäckström and Johansson, 2006; Jamal and Adelowore, 2008). The relationship between the employees and the brand, the so-called behavioral branding, is of particular importance (Manarioti and Kaufmann, 2014). Behavioral branding is defined as employee behavior resulting in an interaction with customers in order to increase their positive attitude toward the brand (Mazzei and Ravazzani, 2015). Consequently, employees’ behavior is expected to be brand consistent (Henkel et al., 2007). The aim is to avoid cognitive dissonance regarding how consumers experience the brand (Cummings and Venkatesan, 1976; Powers and Jack, 2013). Cognitive dissonance can be created after being confronted with conflicting information (Oshikawa, 1969). This discrepant impression can occur through the inappropriate behavior of employees representing the brand. The information they provide is not on-brand; inevitably, this will lead to clients having a less positive opinion about the brand and its equity.

In the marketing literature, brand equity is a pivotal construct and has important implications for brand management (Farquhar, 1989; Aaker and Keller, 1990; Barwise, 1993; Keller, 2003). The so-called Consumer-based Brand Equity (CBBE) has been defined as the incremental utility of a branded product in comparison to its unbranded counterpart (Leuthesser, 1988; Aaker, 1991; Keller, 1993, 2003). Customer satisfaction, brand loyalty, and the brand’s ability to command a markup are positively influenced by high brand equity (Aaker, 1991, 1996; Park and Srinivasan, 1994).

Brand personality is one of the drivers of CBBE (Keller, 1993). Aaker (1997), p. 347 defined brand personality as “the set of human characteristics associated with a brand.” Because consumers perceive brands as extensions of themselves, they associate human personality traits with brands (Belk, 1988). Human personality has been described in many ways, the most prominent of which is the “Big Five” factor structure (Goldberg, 1990; John and Srivastava, 1999). A similar factor structure can be found in Aaker’s (1997) dimensions of brand personality. What could be more obvious than measuring the quality of behavioral branding by examining the extent to which the employee’s behavior, which expresses his or her personality during a sales encounter, is sending out contradictory signals about the brand’s personality?

This article discusses the results of an empirical study with 48 different sales encounters by nine employees for six brands. In doing so, it explains the mechanism of how a brand ambassador’s behavior influences consumers’ reception of the brand and how this can affect brand equity. While the effects of behavioral branding might be understood as a rather intuitive fit between the employee and the brand, the terms, personality and equity, are suitable to operationalize behavioral branding mechanisms. In addition to making a theoretical contribution to the field, this article provides helpful advice for practitioners.

The rest of this article is organized as follows. The next section provides a theoretical overview of the topic and presents the four hypotheses formulated for this empirical study. Then, the study’s method and results are presented. In the final section of this article, the study’s conclusions are presented and discussed.

## The Link Between Behavioral Branding and Brand Equity

**FIGURE 1:**
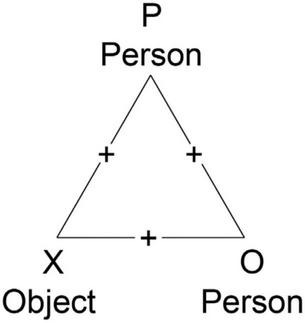
Balanced triad.

Haider’s (1958) balance theory explains how communication influences cognition. The balance theory was developed by Heider (1958) using triads. Triads consist of a person P, a second person O and an object X. Attitudes or factual logical relationships can exist between them. These relationships can be positive or negative in nature. The triad in Figure 1 represents a possible example: P likes pizza (P + X), P is friends with pizza maker O (P + O), O makes the best pizza in town (O + X). In this case, all relationships are positive.

The triads are either in a balanced or in an unbalanced state. The triad in Figure 1 is in balanced. All relationships are positive. In total there exist 2^3^ = 8 triads with different combinations of signs. The status of the triad can be derived from the product of the signs of the individual dyads. If it is positive, the triad is balanced, if it is negative (e.g., if there are two positive and one negative relationship) the triad is unbalanced.

The triads can be used to illustrate the balanced and imbalanced states within a sales process. P becomes the customer, O the salesperson, and X the brand as an object. The interrelationships are P ⇔ X (Consumer’s Attitude toward the brand), P ⇔ O (salesperson’s on-brand behavior during the sales process), and O ⇔ X (employee’s attitude toward the brand). A successful sales process should strive for the one out of the eight possible triads with exclusively positive relationships.

Heider, 1958, p. 176 says that “the concept of a balanced state designates a situation in which the perceived units and the experienced sentiments co-exist without stress.” An unbalanced state produces psychological tension within an individual which causes the so-called cognitive dissonance (Festinger, 1957) where people may feel surprise, dread, guilt, anger, or embarrassment. Due to the negative effects of cognitive dissonance consumer loyalty decreases, negative word-of-mouth increases, the attitude toward the brand is deteriorating and a loss of trust and a subsequent erosion of the brand will follow (Oshikawa, 1969; Cummings and Venkatesan, 1976; Henkel et al., 2007; Bolia et al., 2021). Brand loyalty, brand trust, positive word-of-mouth as positive attitudes toward the brand are all part of brand equity (Keller, 1993, 2009; Brady et al., 2008). Thus, cognitive dissonance induced by an imbalanced state due to non-adequate behavior decimates brand equity. This might be the case when the customer’s attitude toward the brands is positive and also the salesperson’s attitude toward the brand is positive, however, the salesperson’s behavior does not fit to the brand. As an example, this might be the case when a customer likes a Ferrari car and also the salesperson likes Ferrari, however, the salesperson is unable to demonstrate the car because he or she has no driving license. This fairly simple example shows how dissonance can occur and may subsequently damage the brand. On the other hand, positive behavioral branding increases brand equity.

First and foremost, humans are persons. The derivation of the word, person, seems to be controversial. Some scholars assign it to the Latin word, *persona*, which means mask or character. Persona has also been used to refer to the “sounding through” (*personare* = to sound through) of an actor’s voice through his mask. A person’s behavior exhibits his or her individual characteristics, which are called his/her “personality.” An individual’s personality is mostly stable over time and may “sound through” even without engaging in an activity. Thus, it is only one part of human behavior. The other part of human behavior is the way in which a person communicates with words or actions, such as occurs during a sales encounter. To evaluate a salesperson’s behavior, both the salesperson’s personality and his/her performance in the context of a sales encounter will be analyzed.

Since masculinity and femininity as human personality traits are relevant to brands as well as to people (Aaker, 1997; Grohmann, 2009), the personality of a salesperson can be measured using brand gender scales, as proposed and verified by Grohmann (2009). Previous research has demonstrated that the stronger the brand gender attributes, the stronger the brand equity (Lieven et al., 2014, 2015; Tilburg et al., 2015; Lieven and Hildebrand, 2016). Thus, the study tested the following hypothesis:

H1: The stronger the gender dimensions of brand personality, the stronger the brand’s equity.

Similarly, a salesperson’s personality measured by gender dimensions strengthens the fit to the brand and increases its equity; thus:

H2: The more the employee’s personality outperforms (underperforms) a brand’s personality, the more brand equity is strengthened (weakened).

In addition to these personality traits, the sales encounter experience will affect brand equity; thus:

H3: The more (less) adequate a sales encounter, the more brand equity is strengthened (weakened).

It could be argued that the impression of the sales encounter is essential to the attitude toward the brand, so personality is irrelevant. Here, it is also suspected that the sales encounter plays a more important role, because personality is expressed during the encounter. This is done by mediating the personality through the sales encounter, thus:

H4: The effect of the difference between the salesperson’s personality and the brand personality is mediated by the experience of the sales encounter.

These hypotheses were tested in two subsequent surveys.

## Empirical Study

### Methods

This study analyzed the personalities of employees during a sales encounter as a result of their behavioral branding to determine if their personalities matched the brand personalities and whether any discrepancies in personalities cause a change in brand equity. Toward that end, Grohmann’s (2009) gender dimensions of brand personality was applied. This scale has been verified several times and it was found to be valid for both brands and humans, reproducible and invariant across different countries and cultures (Grohmann, 2009; Lieven et al., 2014; Tilburg et al., 2015; Lieven and Hildebrand, 2016; Lieven, 2018). The results showed that strong brand gender increases brand equity. Brand equity was measured according to Brady et al. (2008). This brand equity scale was found to be sufficiently reliable (α = 0.86, Voorhees et al., 2006; α = 0.84, Brady et al., 2008) and valid [average variance extracted (AVE) = 0.55, Voorhees et al., 2006].

However, a crucial problem occurs with the type of analysis described above. In this study, brand equities have to be assessed twice, once before the sales encounter and once after. In case, this is done by the same survey participant this could result in common methods bias or source bias (Podsakoff et al., 2003). Scores for brand equity before the sales encounter could bias the equity scores stated after the encounter. Consequently, the first assessment of brand equity and the brand gender personality were moved to a separate survey.

Thus, the ratings for the employees’ personality after a sales encounter and the brand equity ratings were assessed in a second survey. Thereafter, the brand equity ratings and the employee ratings were compared before and after the encounter and the brand equity ratings were also compared before and after the encounter.

The surveys were conducted online in Germany. The participants were invited to take the online surveys via email; the participants were chosen by an international service provider for online research. Participants were selected according to the rule that the entire sample should follow Germany’s gender and age distribution as closely as possible.

### Study 1: Brand Personality and Brand Equity Before the Sales Encounter

Six well-known brands in Germany were presented to the survey participants: NIVEA (cosmetics), C&A (chain of clothing stores), Canon (cameras), VW (automobile), LUFTHANSA (airline), and SPARKASSE (savings bank). These represent a variety of brands with tangible and non-tangible products and services. Three of the six brands were chosen randomly for each respondent. First, Grohmann’s (2009) 12 personality items were assessed on a 5-point Likert scale, ranging from 1 = does not apply at all to 5 = does fully apply. Thereafter, the five equity scores (Brady et al., 2008) were rated by the participants on a 7-point Likert scale using the semantic differential of the respective item. As another *a priori* measure, the visual brand fit of nine salespersons according to the six brands was assessed. The participants had to rank the employees from 1 to 9. From the average rankings, the ratings were calculated, ranging from 9 points (highest ranking) to 1 point (lowest ranking).

#### Study 1 Results

A total of 507 respondents participated in Study 1 (51.7% female, *M*_*Age*_ = 42.8 years, *SD*_*Age*_ = 13.6 years). Since each respondent answered questions about three randomly selected brands out of the six possible brands, about 1,500 brand evaluations were received, which resulted in an average of 250 ratings for each brand. The brand gender items were aggregated to the masculine brand personality (MBP) and the feminine brand personality (FBP), respectively, and the five items of the Brady et al. (2008) were aggregated to brand equity (Tables 1A,B).

**TABLE 1:** (A) Brand personalities before the sales encounter (5-point Likert scale).

Brand	*N*	Adventurous	Aggressive	Brave	Daring	Dominant	Sturdy	Masculine brand personality (MBP)	Expresses tender feelings	Fragile	Graceful	Sensitive	Sweet	Tender	Feminine brand personality (FBP)
NIVEA	238	3.02	2.66	2.97	3.16	2.96	3.48	3.04	3.34	2.71	3.21	3.36	3.29	3.52	3.24
C&A	238	2.91	2.61	2.90	2.95	2.71	3.24	2.89	2.76	2.62	2.94	2.84	3.01	3.05	2.87
CANON	247	3.18	2.76	3.08	3.19	3.02	3.35	3.08	2.74	2.69	3.00	2.92	2.93	3.00	2.87
VW	262	3.15	2.91	3.15	3.20	3.17	3.45	3.17	2.68	2.65	3.06	2.77	2.91	2.88	2.83
LUFTHANSA	250	3.25	2.87	3.10	3.18	3.16	3.29	3.13	2.76	2.73	3.09	2.92	2.97	2.93	2.90
SPARKASSE	267	2.70	2.70	2.86	2.95	2.97	3.30	2.92	2.53	2.60	2.68	2.75	2.73	2.72	2.66
Total	1502	3.03	2.75	3.01	3.11	3.00	3.35	3.04	2.79	2.66	2.99	2.92	2.97	3.01	2.89

(B) Brand equities before the sales encounter (7-point Likert scale).							

**Brand**	***N***			**Not loyal – very loyal**			**Negative attitude – positive attitude**		**Negative image – positive image**		**Low quality – high quality**		**No willingness – high willingness to pay more**		**Brand equity**	

NIVEA	238			5.07			5.37		5.57		5.46		4.44		5.19	
C&A	238			4.26			4.72		4.72		4.71		3.59		4.40	
CANON	247			4.51			4.83		5.05		5.13		4.36		4.77	
VW	262			4.48			4.74		4.69		5.04		4.03		4.59	
LUFTHANSA	250			4.45			4.79		4.95		5.13		4.20		4.70	
SPARKASSE	267			4.55			4.60		4.68		4.74		3.59		4.44	
Total	1502			4.55			4.83		4.93		5.03		4.03		4.67	

The brand personalities and brand equities differed significantly across the six brands [*F*(5,1496) = 4.142, *p* < 0.001 for MBP; *F*(5,1493) = 10.778, *p* < 0.001 for FBP; *F*(5,1502) = 10.856, *p* < 0.001 for brand equity]. Similar to previous studies (Lieven et al., 2014; Lieven and Hildebrand, 2016), brand gender (as brand personality) increased brand equity. In a linear regression, MBP increased brand equity by 0.331, FBP increased brand equity by 0.293, and the interaction of MBP × FBP increased brand equity by 0.079 (all *p*’s < 0.001, *R*^2^ = 0.342). This supports H1.

The ratings for the visual brand fit of nine salespersons are shown in Table 2. The higher the ratings, the better the perceived fit to the respective brand. In general, female salespersons seem to have a better brand fit to traditionally feminine brands (NIVEA and C&A) and male salespersons seem to have a better brand fit to traditionally masculine brands (CANON and VW). For LUFTHANSA and SPARKASSE, the brand ratings are more equally distributed since these brands are neither masculine nor feminine (Lieven, 2018).

**TABLE 2:** Average ratings for the visual brand fit of nine employees (highest rating = 9).

Employee	1	2	3	4	5	6	7	8	9
	Female				Male				
NIVEA	6.22	7.37	6.56	6.17	3.09	3.16	3.63	5.39	4.66
C&A	6.40	7.21	6.33	5.66	3.47	3.73	4.07	5.19	4.56
CANON	5.07	5.79	5.37	5.28	4.13	4.70	5.20	6.91	4.73
VW	4.34	4.96	4.53	5.02	5.71	5.09	6.27	6.99	4.83
LUFTHANSA	4.90	6.04	4.99	5.19	4.99	4.10	5.24	6.59	4.29
SPARKASSE	5.44	5.40	4.73	5.81	5.13	4.23	5.27	6.19	4.87

### Study 2: Employee Personality and Brand Equity After the Sales Encounter

In 48 different scenarios, the six brands were presented to the survey participants combined with one picture of the salespersons shown in Table 2 and an audio file with an oral sales encounter. The encounters differed regarding the salesperson’s competence and friendliness. The wording and the prosody (i.e., rhythm, stress, and intonation of speech) were manipulated on a convenience level. The aim was to create a collection of everyday encounters that consumers could have experienced^1^. In order to control for the effects of unkindness or incompetence, different sales encounters were applied to the same brand and the same salesperson where only the behavior was manipulated, respectively.

The respondents were first asked to rate the respective employee’s personality based on Grohmann’s (2009) gender personality model. Then, they had to rate their impression of the sales encounter in relation to courtesy, competence, attractiveness, and friendliness. Thereafter, the respondents rated their attitude to the respective brand based on Brady et al. ’s (2008) equity model.

#### Study 2 Results

A total of 3,824 respondents participated in Study 2 (52.9% female, *M*_*Age*_ = 43.0 years, *SD*_*Age*_ = 12.9 years). Each respondent rated randomly four of the 48 sales encounters, which resulted in an average of more than 300 ratings per scenario (a total of 15,296 evaluations). The scores were aggregated to masculine and feminine employee personalities, sales encounter experience, and brand equity after the sales encounter. To determine the shift from the baseline scores from Study 1 to the actual scores after the sales encounter in Study 2, the respective personality and equity scores were subtracted. The results are presented in Table 3, which give a first impression that the personality difference and the sales encounter correlate with the shift in brand equity. When the sales encounter experience is above average and the difference in personality is positive, the brand equity increases.

**TABLE 3:** Brand equities before and after 48 sales encounters.

Encounter	*N*	Brand	Sales person	Visual brand Fit	Masculine brand personality	Feminine brand personality	Brand equity before sales encounter	Masculine employee personality	Feminine employee personality	Encounter experience	Brand equity after sales encounter	Personality difference	Difference in brand equity
1	346	NIVEA	1	6.22	3.04	3.24	5.18	2.91	3.05	4.84	4.78	−0.32	−0.41
2	322	NIVEA	1	6.22	3.04	3.24	5.18	3.00	2.42	3.65	4.11	−0.86	−1.07
3	300	NIVEA	2	7.37	3.04	3.24	5.18	2.85	3.13	5.02	4.79	−0.30	−0.39
4	338	NIVEA	2	7.37	3.04	3.24	5.18	2.89	3.11	5.12	4.93	−0.28	−0.25
5	284	NIVEA	4	6.17	3.04	3.24	5.18	3.07	2.70	4.45	4.50	−0.51	−0.69
6	349	NIVEA	4	6.17	3.04	3.24	5.18	3.02	2.42	3.36	3.97	−0.83	−1.22
7	293	NIVEA	6	3.16	3.04	3.24	5.18	3.03	2.64	4.07	4.41	−0.61	−0.77
8	309	NIVEA	6	3.16	3.04	3.24	5.18	3.00	2.33	3.21	3.98	−0.95	−1.20
9	337	C&A	2	7.21	2.89	2.87	4.40	2.84	3.01	5.07	4.59	0.10	0.19
10	311	C&A	1	6.40	2.89	2.87	4.40	2.92	2.43	3.52	3.46	−0.41	−0.94
11	323	C&A	4	5.66	2.89	2.87	4.40	3.03	2.83	4.73	4.44	0.11	0.04
12	312	C&A	6	3.73	2.89	2.87	4.40	3.03	2.35	3.43	3.65	−0.38	−0.75
13	329	C&A	9	4.56	2.89	2.87	4.40	2.80	3.04	4.84	4.52	0.08	0.12
14	303	C&A	7	4.07	2.89	2.87	4.40	2.68	2.17	3.00	3.28	−0.91	−1.12
15	318	CANON	2	5.79	3.10	2.88	4.78	2.89	3.01	5.03	4.75	−0.08	−0.02
16	306	CANON	2	5.79	3.10	2.88	4.78	2.90	3.06	5.18	4.84	−0.01	0.06
17	318	CANON	4	5.28	3.10	2.88	4.78	2.99	2.94	5.07	4.84	−0.05	0.07
18	311	CANON	6	4.70	3.10	2.88	4.78	2.98	2.49	4.01	4.24	−0.51	−0.54
19	315	CANON	7	5.20	3.10	2.88	4.78	2.91	2.88	5.05	4.85	−0.18	0.07
20	311	CANON	8	6.91	3.10	2.88	4.78	2.96	2.76	4.78	4.67	−0.26	−0.10
21	328	CANON	9	4.73	3.10	2.88	4.78	2.81	2.93	5.00	4.79	−0.24	0.02
22	325	VW	1	4.34	3.17	2.83	4.60	3.03	2.33	3.52	3.70	−0.64	−0.90
23	329	VW	2	4.96	3.17	2.83	4.60	2.90	3.14	5.18	4.62	0.05	0.02
24	331	VW	4	5.02	3.17	2.83	4.60	3.03	2.80	4.64	4.43	−0.17	−0.16
25	307	VW	3	4.53	3.17	2.83	4.60	2.95	3.03	5.09	4.65	−0.01	0.05
26	342	VW	5	5.71	3.17	2.83	4.60	2.72	2.60	4.25	4.09	−0.68	−0.51
27	299	VW	8	6.99	3.17	2.83	4.60	2.86	2.76	4.80	4.52	−0.38	−0.07
28	321	VW	7	6.27	3.17	2.83	4.60	2.89	2.15	3.04	3.32	−0.96	−1.27
29	352	VW	6	5.09	3.17	2.83	4.60	3.07	2.52	3.99	4.02	−0.40	−0.57
30	309	VW	9	4.83	3.17	2.83	4.60	2.73	2.87	4.77	4.43	−0.40	−0.17
31	304	LUFTHANSA	1	4.90	3.14	2.90	4.70	2.90	2.25	3.12	3.33	−0.89	−1.38
32	317	LUFTHANSA	5	4.99	3.14	2.90	4.70	2.72	2.24	3.28	3.44	−1.08	−1.27
33	308	LUFTHANSA	6	4.10	3.14	2.90	4.70	2.96	2.47	3.72	3.93	−0.61	−0.77
34	326	LUFTHANSA	9	4.29	3.14	2.90	4.70	2.69	2.96	5.01	4.76	−0.39	0.06
35	326	LUFTHANSA	8	6.59	3.14	2.90	4.70	2.83	2.92	5.06	4.77	−0.29	0.07
36	313	LUFTHANSA	2	6.04	3.14	2.90	4.70	2.91	3.23	5.52	4.87	0.10	0.17
37	321	LUFTHANSA	4	5.19	3.14	2.90	4.70	2.95	3.09	5.22	4.78	0.00	0.08
38	296	LUFTHANSA	3	4.99	3.14	2.90	4.70	2.86	3.07	5.22	4.72	−0.11	0.01
39	302	SPARKASSE	4	5.81	2.91	2.67	4.43	2.89	2.68	4.57	4.21	−0.02	−0.22
40	341	SPARKASSE	2	5.40	2.91	2.67	4.43	2.97	3.06	4.98	4.40	0.45	−0.03
41	334	SPARKASSE	3	4.73	2.91	2.67	4.43	2.90	2.87	5.03	4.47	0.19	0.04
42	320	SPARKASSE	1	5.44	2.91	2.67	4.43	2.90	2.31	3.57	3.44	−0.37	−0.99
43	325	SPARKASSE	5	5.13	2.91	2.67	4.43	2.87	2.76	4.75	4.34	0.05	−0.09
44	305	SPARKASSE	5	5.13	2.91	2.67	4.43	2.91	2.48	3.95	3.81	−0.19	−0.62
45	320	SPARKASSE	6	4.23	2.91	2.67	4.43	2.90	2.26	3.48	3.55	−0.42	−0.89
46	336	SPARKASSE	8	6.19	2.91	2.67	4.43	2.86	2.68	4.50	4.13	−0.04	−0.30
47	289	SPARKASSE	7	5.27	2.91	2.67	4.43	2.85	2.65	4.56	4.21	−0.08	−0.23
48	335	SPARKASSE	7	5.27	2.91	2.67	4.43	2.94	2.25	3.30	3.29	−0.39	−1.14

**FIGURE 2:**
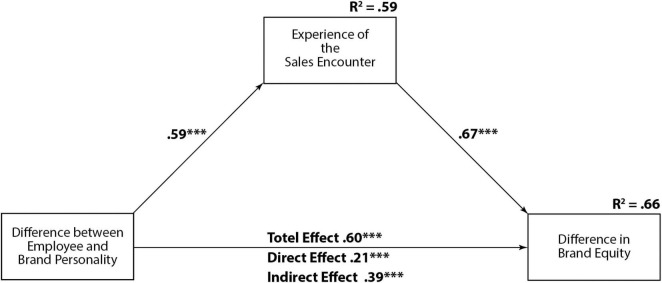
Mediation model. ***p < 0.001.

For a more in-depth analysis, all 15,296 ratings were evaluated with a linear regression of personality difference as the independent variable and equity change as the outcome variable. The standardized coefficient was positive and significant 0.60 (*p* < 0.001), which supports H2. Thus, the more negative the difference between the employee’s and the brand’s personality, the greater the loss in brand equity, and vice versa. Performing the same regression with the sales encounter experience instead of personality change also resulted in a significant positive coefficient, which supports H3 (0.794, *p* < 0.001). Both the sales encounter experience and the difference in brand and salesperson personality contribute to the change in brand equity. To ascertain the importance of both variables on the equity change, a model with the sales encounter experience as a mediator was evaluated. This resulted in a direct effect of personality change of only 0.21 instead of the total effect of 0.60 (*p* < 0.001). Consequently, as seen in Figure 2, the encounter experience mediated personality difference by an indirect effect of 0.59 × 0.67 = 0.39 (*p* < 0.001). Thus, H4 is supported as well.

To ascertain the role of the visual brand fit of the employee to the brand, linear regression resulted in a small, but positive, effect of 0.07, *p* < 0.001, *R*^2^ = 0.0049. The high significance is due to the high number of cases: 15,296. Calculating Cohen’s *f* (Cohen, 1988; Selya et al., 2012) to assess the effect size of visual fit resulted in a low value of 0.0049/(1–0.0049) = 0.005, which is well below 0.02 where the effects start to have at least a small effect (Cohen, 1988). Including the visual brand fit in the above model as a further mediator resulted in a small increase in the coefficient of determination from 0.6581 to 0.6597; thus, the *a priori* visual brand fit has no influence on behavioral branding. As a comparison, inclusion of the sales encounter as a mediator increases the R^2^ of the outcome variable with a very large effect size [Cohen’s *f* = (0.66–0.36)/(1–0.66) = 0.88].

## Conclusion

This study aimed to demonstrate how adequate or inadequate behavioral branding affects brand equity. While the evaluation of behavior is mostly intuitive, this study operationalized behavior using two components: personality and the sales encounter experience. The construct of gender allowed for a comparison of the salesperson’s personality and the brand’s personality. The more the employee’s personality outperformed a brand’s personality, the more brand equity was strengthened, and vice versa. The sales encounter experience had a stronger effect; however, it did not suppress the personality effect, it partly mediated it.

As seen in the results presented in Table 3, in some cases, it was only possible to increase the *a priori* brand equity. That means that behavioral branding should attempt to maintain the level of the existing equity. It is more likely that consumers will experience cognitive dissonance as a result of conflicting information regarding the brand rather than being positively surprised by adequate behavioral branding.

The choice of gender as a personality model was done consciously. There are other personality models (Aaker, 1997; Geuens et al., 2009), but they are increasingly being criticized for not being reproducible or for not being able to be applied to both people and brands (Caprara et al., 2001; Austin et al., 2003). The Grohmann (2009) gender model turned out to be well-suited to describing humans and brands [for an in-depth analysis see Lieven (2017, 2018)]. As well, there might be more components that influence brand equity than only personality and sales encounter. However, these two seemed to be the most suitable to be measured in a quantitative study.

The results were not differentiated according to masculine or feminine brands and employees. Lieven and Hildebrand (2016) and Lieven (2018) have shown that the simultaneous increase in masculinity and femininity increases brand strength even further. It may then be androgynous brands that are among the strongest (in this sample, Nivea could be such an androgynous brand). For this reason, the summed-up gender characteristics of the employees were compared with those of the summed-up brand genders.

All the coefficients of the regression model were highly significant. This is not surprising with the large sample size of Study 2 since a larger number of cases increases the significance. However, what is surprising is the high degree of explained variance, which tends to diminish in larger samples. Overall, two-thirds of the brand equity changes are explained by the mediation model (*R*^2^ = 0.66).

The problem of common method bias or source bias (Podsakoff et al., 2003) could be solved at least for the brand equities since these were assessed as baseline scores without the sales encounter, and separately before and after the sales encounter in different surveys. Thus, the brand equity evaluations before the sales encounter could not bias the equity evaluations after the encounter. A single factor test (Harman, 1976) for the data of Study 2 resulted in 52.9% of explained variance, which is slightly above the threshold of 50%. However, a three-factor solution showed differentiated constructs for the masculine and feminine personalities. The third factor consisted of the sales encounter experience and the brand equity, which caused the high explained variance. However, as has been shown, brand equity was strongly affected by the sales encounter experience, and a high consistency between both constructs is not surprising. Thus, unwanted bias did not influence equity; rather, equity was affected by the outcome of the sales encounter.

The demonstration of the impact of behavioral branding on brand equity is a strong theoretical contribution of this study. However, results may be even more important for practitioners. The findings provide opportunities to control brand awareness, including the training of their brand ambassadors which might become as same as important as technical or competence training. The personality concept can help to increase the fit between the brand and the employee since both brands and humans can be endowed with personality. Managing brand personality thus also means managing employees’ personality.

As often in scientific research the experimental designs were hypothetical. Studies conducted with real-world salespersons would be a promising venue for further research.

## Participants

Human survey respondents in this study were randomly invited to participate on a voluntary basis. There are no identifiable images or data in the manuscript. The images of the stimuli for the sales personal are professional photographies licensed from istockphoto.com.

## Data Availability Statement

The raw data supporting the conclusions of this article will be made available by the authors, without undue reservation.

## Ethics Statement

Ethical review and approval was not required for the study on human participants in accordance with the local legislation and institutional requirements. The patients/participants provided their written informed consent to participate in this study.

## Author Contributions

The author confirms being the sole contributor of this work and has approved it for publication.

## Conflict of Interest

The author declares that the research was conducted in the absence of any commercial or financial relationships that could be construed as a potential conflict of interest.

## Publisher’s Note

All claims expressed in this article are solely those of the authors and do not necessarily represent those of their affiliated organizations, or those of the publisher, the editors and the reviewers. Any product that may be evaluated in this article, or claim that may be made by its manufacturer, is not guaranteed or endorsed by the publisher.

## Footnotes

1 The sales encounters can be downloaded from www.researchfiles.com/01.mp4 where 01 to 48 is the number of the respective scenario 01, 02,…48. Although the language is German, the appropriateness of the encounters can be evaluated by anyone through their prosody (rhythm, stress, and intonation of speech).
